# Masking as an effective quality control method for next-generation sequencing data analysis

**DOI:** 10.1186/s12859-014-0382-2

**Published:** 2014-12-13

**Authors:** Sajung Yun, Sijung Yun

**Affiliations:** John A. Burns School of Medicine, University of Hawai‘i at Manoa, Honolulu, HI USA; Laboratory of Molecular Biology, National Institute of Diabetes and Digestive and Kidney Diseases, National Institutes of Health, Bethesda, MD USA

**Keywords:** NGS, Preprocessing, Masking, Trimming

## Abstract

**Background:**

Next generation sequencing produces base calls with low quality scores that can affect the accuracy of identifying simple nucleotide variation calls, including single nucleotide polymorphisms and small insertions and deletions. Here we compare the effectiveness of two data preprocessing methods, masking and trimming, and the accuracy of simple nucleotide variation calls on whole-genome sequence data from *Caenorhabditis elegans*. Masking substitutes low quality base calls with ‘N’s (undetermined bases), whereas trimming removes low quality bases that results in a shorter read lengths.

**Results:**

We demonstrate that masking is more effective than trimming in reducing the false-positive rate in single nucleotide polymorphism (SNP) calling. However, both of the preprocessing methods did not affect the false-negative rate in SNP calling with statistical significance compared to the data analysis without preprocessing. False-positive rate and false-negative rate for small insertions and deletions did not show differences between masking and trimming.

**Conclusions:**

We recommend masking over trimming as a more effective preprocessing method for next generation sequencing data analysis since masking reduces the false-positive rate in SNP calling without sacrificing the false-negative rate although trimming is more commonly used currently in the field. The perl script for masking is available at http://code.google.com/p/subn/. The sequencing data used in the study were deposited in the Sequence Read Archive (SRX450968 and SRX451773).

## Background

Research and clinical applications of next generation sequencing (NGS) have been widely accepted as useful and sensitive approaches for detecting genetic variants. Accurate base calling is key to this discovery tool because both false-negative and false-positive calls may complicate the interpretation of the data with respect to causative mutations. However, the raw data obtained from NGS is known to contain some low quality base calls for which no consensus has been reached about their proper interpretation. Bioinformatic quality control methods have been introduced into NGS analysis pipeline to increase the accuracy of simple nucleotide variation (SNV) calls that include single nucleotide polymorphism (SNP) and insertion and deletion (indel).

Trimming is a commonly used bioinformatic quality control method for base calls with low quality. It deletes base calls in a NGS read such that there remains a contiguous string of bases with quality scores above a user defined cutoff threshold or until the average quality of the remaining reads falls below the threshold value. The software programs available for trimming include Btrim [1], ConDeTri [2], FASTQ quality trimmer in Galaxy [3], SeqTrim [4], and SolexaQA [5]. In contrast, masking substitutes low quality base calls with an ‘N’, an undetermined base though it is not widely used compared to trimming. Software programs available for masking include FASTQ Masker in Galaxy [3], and FASTX Toolkit [6]. Recently, Liu et al. showed that trimming introduced lots of false-positives and they noted that it is necessary to have more efficient bioinformatics algorithm for NGS data preprocessing [7]. Here we evaluated the effectiveness of trimming and masking by performing DNA whole-genome sequencing in *Caenorhabditis elegans* (*C. elegans*). The analysis showed that masking reduced the false-positive rate without increasing the false-negative rate with statistical significance. However, trimming was not as effective as masking in reducing the false-positive rate.

## Methods

### Experimental design

To evaluate the false-positive rate, we performed DNA-seq on a *C. elegans* strain that was mutagenized by ethyl methanesulfonate (EMS) and then verified the DNA-seq prediction by Sanger sequencing. To evaluate the false-negative rate, we sequenced a Hawaiian *C. elegans* strain and verified our SNP predictions by comparing them to the publicly available Hawaiian SNP data from Wormbase (WS220) [8]. This allowed us to compare the performance of trimming vs. masking to set a standard for quality control in next generation sequencing data analysis.

### Sequencing

**Figure 1:**
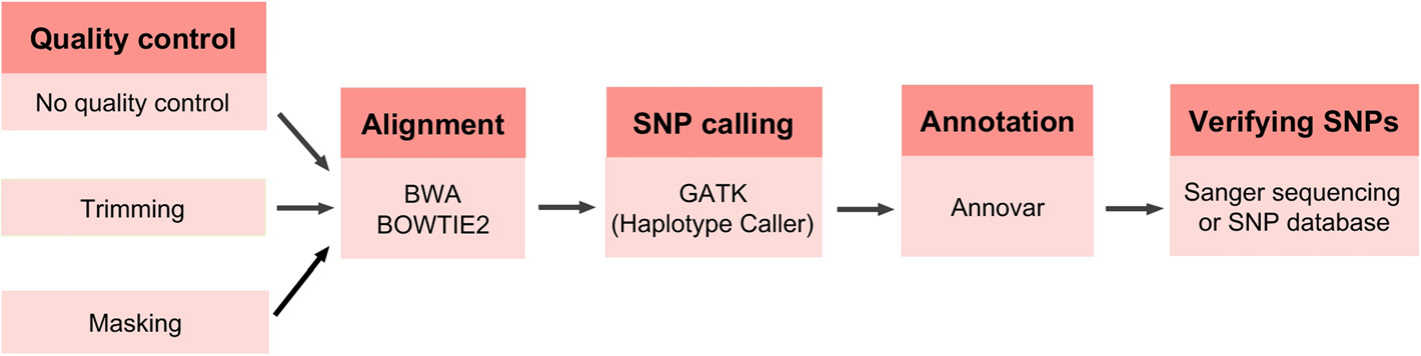
**A schematic diagram for data analysis pipeline.**

We performed 76 cycle single-end sequencing on the *C. elegans* samples using Illumina’s HiSeq 2000. We obtained 27,528,260 reads and 49,699,895 reads for the EMS mutagenized and the Hawaiian strain, respectively, after the default filtering. These corresponded to ~22 fold and ~39 fold genome coverage, respectively. A schematic diagram for data analysis pipeline is shown in Figure 1.

### Preprocessing and alignments

Raw reads were trimmed or masked using SolexaQA [5] and SubN [9] for each. SubN was developed in house, available for public, simple and small (less than 200 lines), and easy to use if PERL is pre-installed. Raw, trimmed, or masked FASTQ files were aligned to the *C. elegans* genome, version WS220, using BWA (version 0.6.2-r126) [10] or BOWTIE2 (version 2.1.0) [11] with default parameters as indicated in the publications cited. We used two aligners to address the dependence of SNV calling on a particular choice of an aligner.

### Variant calling

SNV calling was performed with the haplotype caller in GATK (version2.7-2) package [12,13] and ANNOVAR (version 2011-11-20) [14] was used to annotate missense SNPs. We focused on missense SNPs in evaluating the accuracy of SNP calling because they may have immediate biomedical applicability. For the EMS mutagenized strain, we found 172 ~ 221 homozygous missense SNPs, depending on the aligner and the quality control method used. Then, we checked the SNP and indel predictions to Sanger sequencing result; we performed Sanger sequencing on 145 SNV candidates in exonic region, and obtained 37 positive SNPs, 86 negative SNPs, and 22 Sanger verified indels. Variant calling has a strong dependence on program parameter settings as well as the interfacing tools in the entire analysis pipeline, as recently reported by both O’Rawe et al. and Yu et al. [15,16]. Surveying the influence of all possible combinations of parameter values was beyond the scope of the current study and instead we used the recommended default parameter settings, unless otherwise specified.

### Details on parameters and statistical methods

For the cutoff quality score of trimming or masking, we chose the 1% probability of error, which corresponded to a PHRED score of 20. We used the default value of 25 bases as the minimum length of reads to be kept for trimming. The false-positive rate was calculated as the number of false-positives divided by the total number of validated negatives, i.e. sum of false-positives and true-negatives. To decide whether these differences are statistically significant for the false-positives, we used Fisher’s exact test using R-package (version 3.0.1) [17] based on 2 × 2 contingency table of false-positives and true-negatives. We avoided using χ^2^ test for the false-positives because some values were smaller than five. The false-negative rate was calculated as the number of false-negatives divided by the sum of false-negatives and true-positives. Duplicate removal was performed with MarkDuplicates tool in Picard tools, version 1.75 [18].

## Results and discussion

### Trimming and masking on alignments

**Figure 2:**
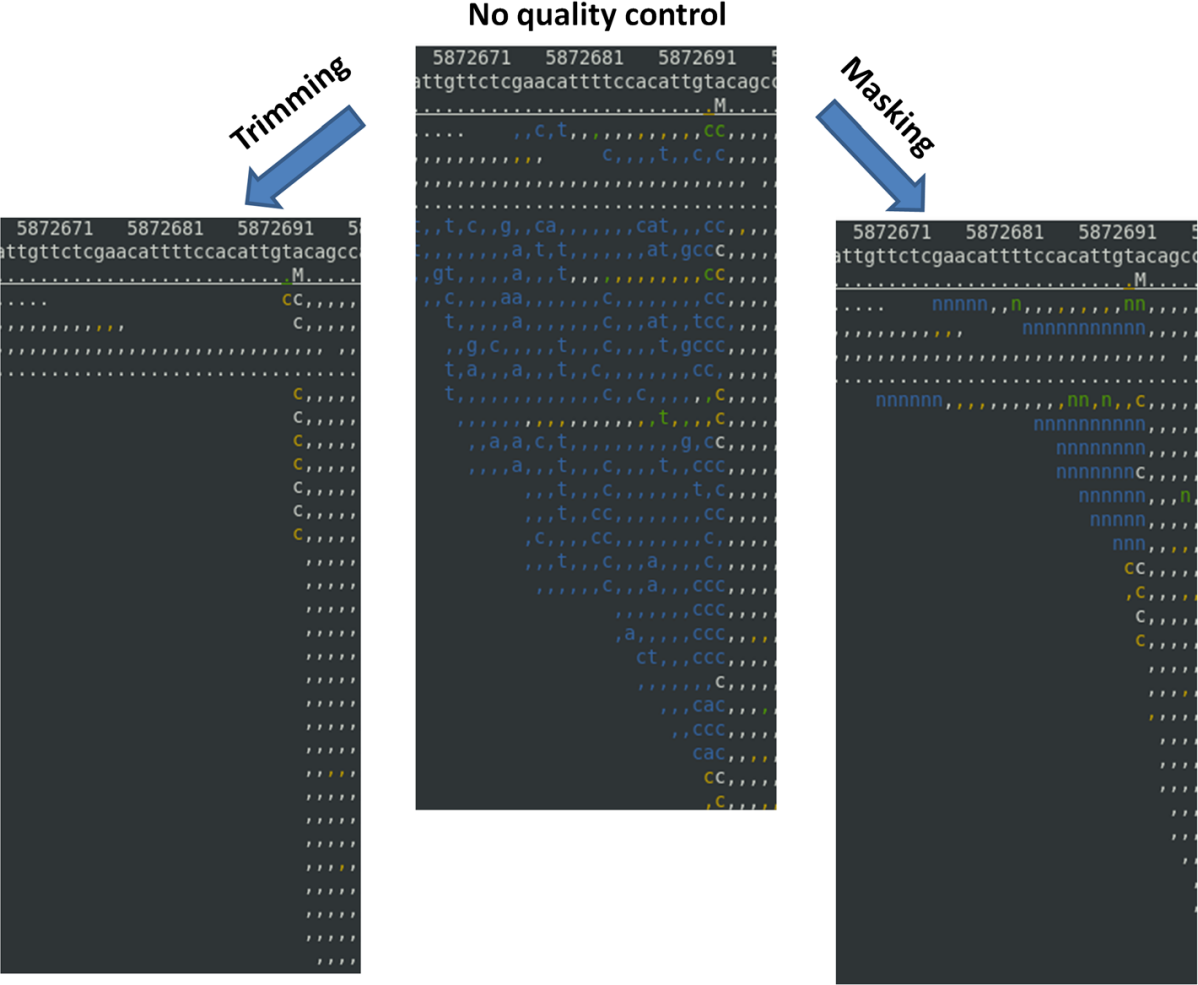
**A typical example of alignments with trimming or masking in respect to no quality control in a region where read quality scores are poor.** The top, the second, and the third line in an alignment picture shows base pair position, the reference genome sequence, and the consensus sequence, for each. A base call that matches the reference sequence is denoted as a dot for the forward strand or as a comma for the reverse strand. The colors in a read denote the base call quality. White is for a base with its error rate smaller than 0.1%, yellow for 0.1 ~ 1% error rate, green for 1 ~ 10% error rate, and blue for 10% or greater error rate. Since we used 1% cutoff, all the green and the blue were either trimmed off or masked as ‘N’ or ‘n’. The graphics were made using tview in Samtools (version 0.1.18) [19]. BOWTIE2 was used to align reads.

The total number of reads was decreased with trimming by 7.1% from 27,528,260 reads to 25,570,162 reads (Table 1). It is because of the dynamic trimming algorithm; first, it identified the longest contiguous stretch of bases whose error rate is smaller than 1% for all the bases. If the number of bases in the stretch was smaller than 25, the read was discarded to avoid the mapping error. However, masking did not change the total number of reads as expected. BWA and BOWTIE2 aligned 93.9% and 97.3% of reads, for each, compared to the total number of raw reads. Trimming resulted in slightly smaller number of reads aligned, 91.2% for BWA and 91.9% for BOWTIE2. However, masking resulted in much smaller number of reads aligned, 82.2% for BWA and 88.7% for BOWTIE2. Aligning more reads that contain low quality base calls is not necessarily desirable because mis-alignments or errors in base calls could result in errors in SNP calling. Figure 2 shows a typical example of alignments on how trimming or masking affects alignment of reads in a region where quality scores are low. A base in a blue or a green color denotes its error rate is higher than 1%. These low quality bases were trimmed off with trimming or masked as ‘n’ with masking.

**Table 1:** **The number of reads and percentage attained after quality control and/or alignment**

**Aligners**	***No quality control***	***Trimming***	***Masking***
	**27,528,260**	**25,570,162 (92.9%)**	**27,528,260 (100%)**
BWA	25,843,686 (93.9%)	25,113,197 (91.2%)	22,617,992 (82.2%)
BOWTIE2	26,784,763 (97.3%)	25,286,787 (91.9%)	24,415,121 (88.7%)

The percentage is calculated to the number reads with no quality control.

### False-positive rate

**Figure 3:**
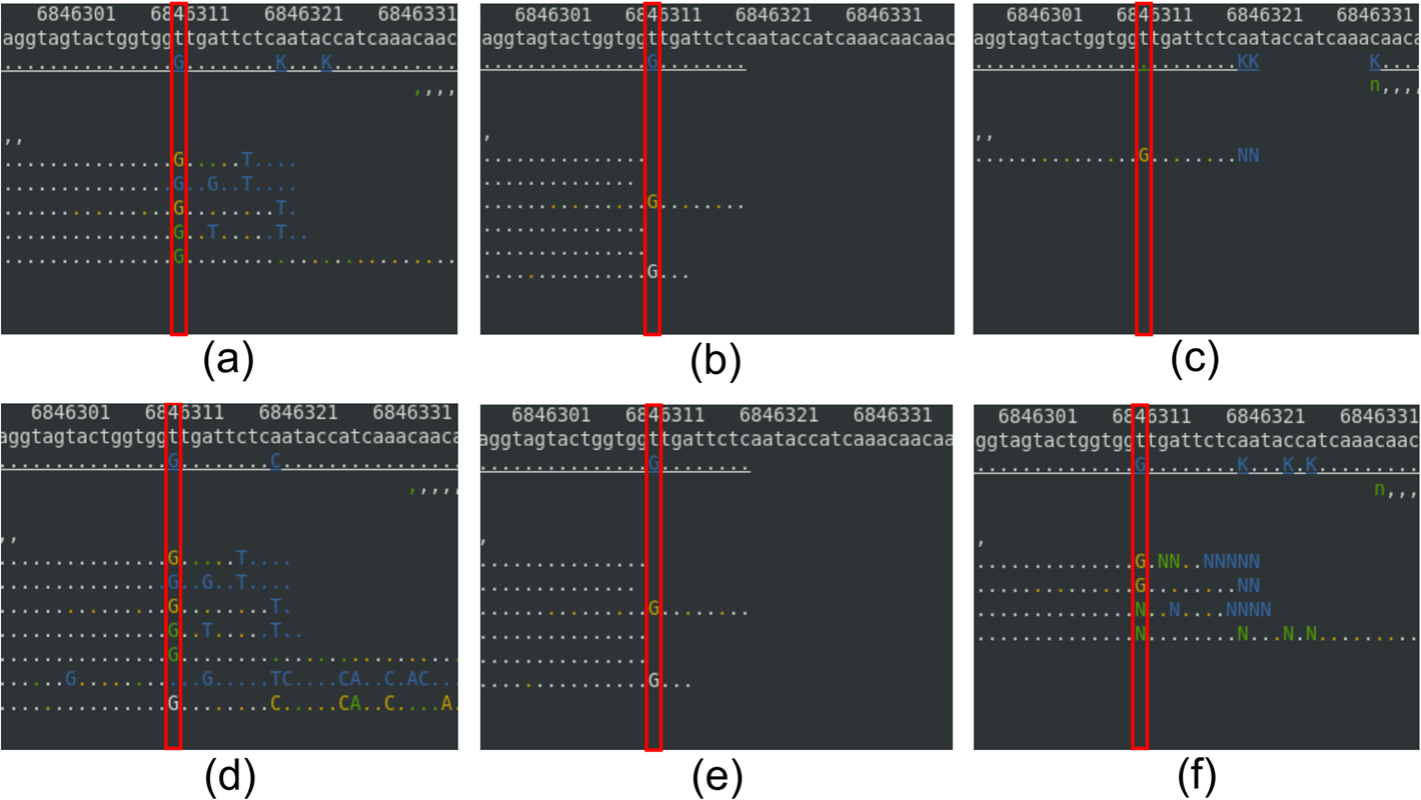
**An example of alignments near a false-positive SNP position with no quality control or with trimming. (a)** no quality control – BWA, **(b)** trimming – BWA, **(c)** masking – BWA, **(d)** no quality control – BOWTIE2, **(e)** trimming – BOWTIE2, and **(f)** masking – BOWTIE2. The top, the second, and the third line in an alignment picture show a base pair position, the reference genome sequence, and the consensus sequence, for each. A base call that matches the reference sequence is denoted as a dot for the forward strand or as a comma for the reverse strand. The colors in a read denote the base call quality. White is for a base with its error rate smaller than 0.1%, yellow for 0.1 ~ 1% error rate, green for 1 ~ 10% error rate, and blue for 10% or greater error rate. Since we used 1% cutoff, all the green and the blue were either trimmed off or masked as ‘N’ or ‘n’. The graphics were made using tview in Samtools (version 0.1.18) [19]. BOWTIE2 was used to align reads. GATK haplotype SNP caller made false-positive homozygous SNP calling in **(a)**, **(b)**, **(d)**, and **(e)**, not in **(c)** and **(f)**.

Masking reduced the false-positive rate in SNP calling compared to no quality control for both BWA and BOWTIE2 (Table 2). The false-positive rate dropped from 2.3% to 0% for BWA, and from 7.0% to 0% for BOWTIE2. The p-values for the null-hypothesis were 0.25 and 0.014 for BWA and for BOWTIE2, for each. Therefore, we concluded that masking reduced the false-positive rate with statistical significance for BOWTIE2. Though false-positives rate was decreased for BWA from 2.3% to 0%, it was not statistically significant enough with our 86 Sanger negative SNPs because the number of false-positives with BWA was already too small with no quality control. However, trimming did not decrease the false-positive rate for BWA. Even for BOWTIE2, the decrease in the false-positive rate with trimming was not statistically significant (p-value 0.14). Hence, we conclude that masking is more effective than trimming in reducing the false-positive rate. Figure 3 shows an example of a false-positive SNP calling at chromosome X: 6,846,313 with no quality control and trimming, but a true-negative SNP calling with masking; no quality control and trimming falsely predicted it as a homozygous SNP whereas masking correctly predicted in both BWA and BOWTIE2; Sanger sequencing confirmed a reference base.

**Table 2:** **Number of true-positives, false-positives, true-negatives, and false-negatives in SNP verification by Sanger sequencing for no quality control vs. trimming vs. masking**

***Aligners***	***No quality control***				***Trimming***				***Masking***			
	**TP**	**FP**	**TN**	**FN**	**TP**	**FP**	**TN**	**FN**	**TP**	**FP**	**TN**	**FN**
BWA	35	2 (2.3%)	84	2	35	2 (2.3%)	84	2	35	0 (0%)	86	2
BOWTIE2	35	6 (7.0%)	80	2	35	2 (2.3%)	84	2	35	0 (0%)	86	2

TP: True-Positive, FP: False-Positive, TN: True-Negative, FN: False-Negative. The value with parenthesis in the FP cell denotes the false-positive rate in percent.

### False-negative rate

**Figure 4:**
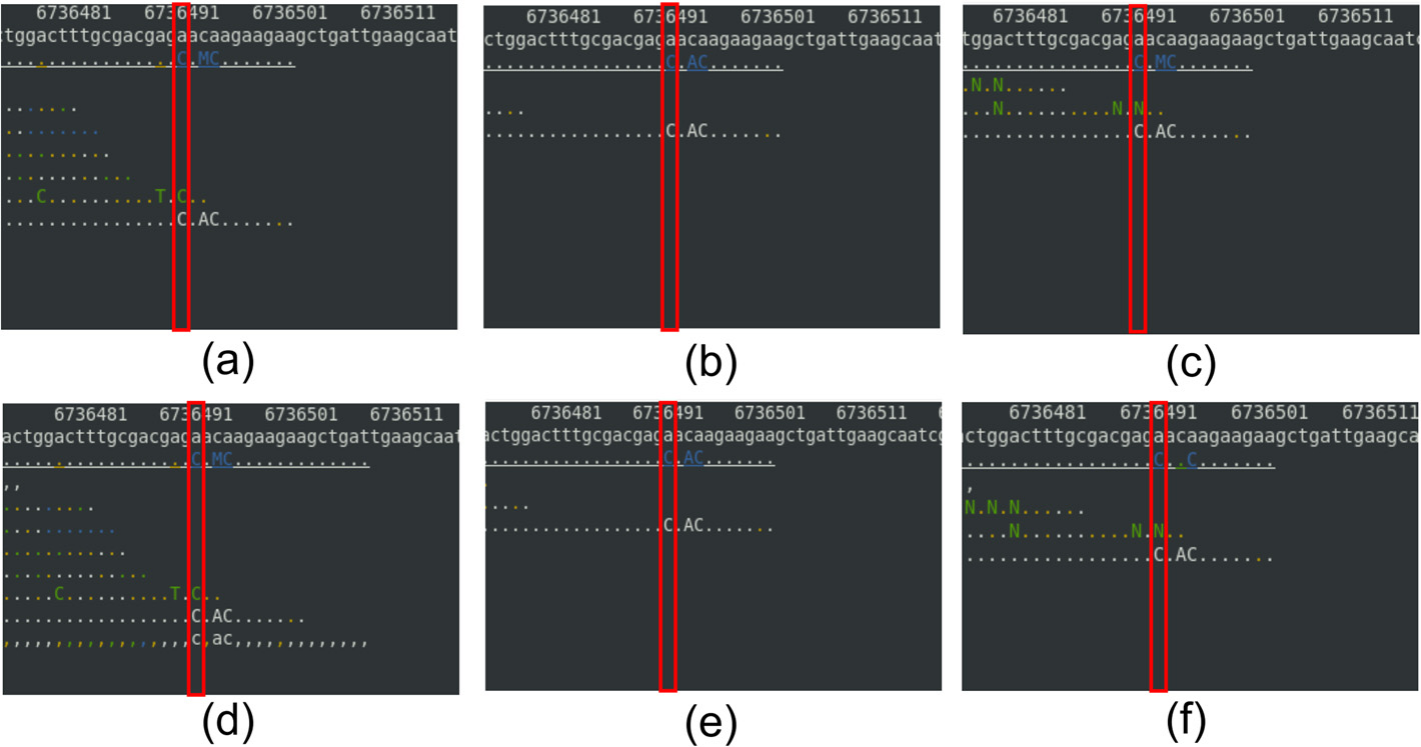
**Alignments for the first false-negative SNP at chromosome II:6,736,494. (a)** no quality control – BWA, **(b)** trimming – BWA, **(c)** masking – BWA, **(d)** no quality control – BOWTIE2, **(e)** trimming – BOWTIE2, and **(f)** masking – BOWTIE2. See the Figure 3 legend for details on coloring.

**Figure 5:**
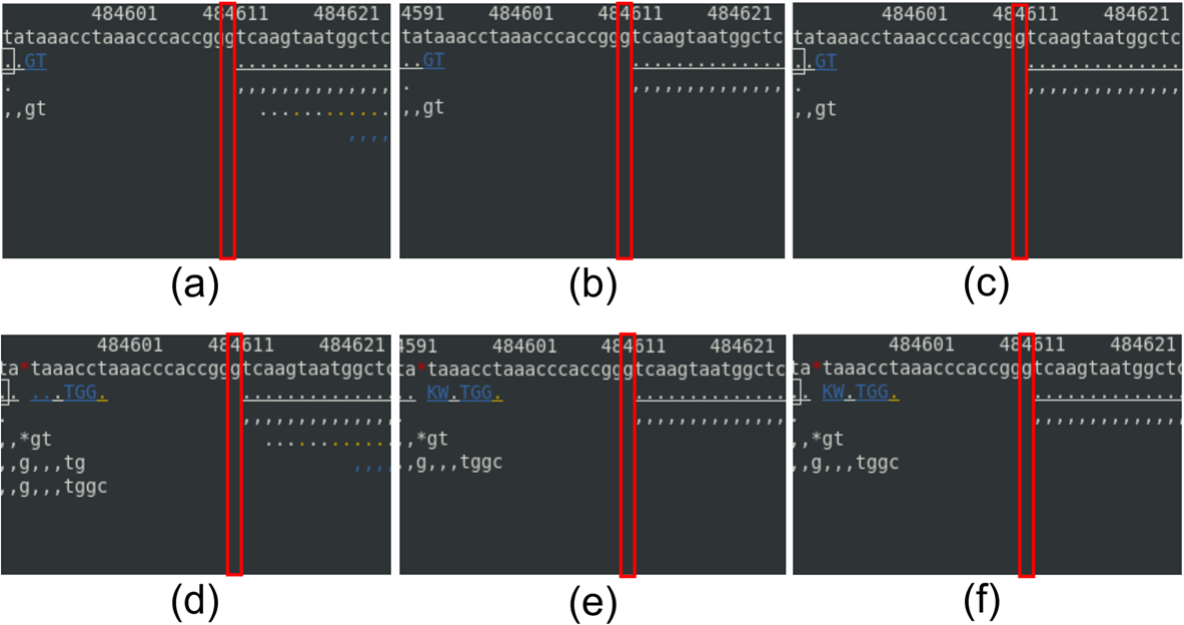
**Alignments for the second false-negative SNP at chromosome X:484,613. (a)** no quality control – BWA, **(b)** trimming – BWA, **(c)** masking – BWA, **(d)** no quality control – BOWTIE2, **(e)** trimming – BOWTIE2, and **(f)** masking – BOWTIE2. See the Figure 3 legend for details on coloring.

Masking and trimming did not improve for the false-negative cases in our SNP (Table 2) and indel cases (Table 3) with the EMS induced mutant strains. There were two false-negative SNPs and two false-negative indels regardless of preprocessing methods and aligners. The two false-negative SNP cases were due to too few reads aligned in the region (Figures 4 and 5) and removing bases of low qualities further did not help in reducing the false-negative rate in SNP calling.

**Table 3:** **The number of true-positives and false-negatives in indel verification by Sanger sequencing for no quality control vs. trimming vs. masking**

***Aligners***	***No quality control***		***Trimming***		***Masking***	
	**True- positive**	**False- negative**	**True- positive**	**False- negative**	**True- positive**	**False- negative**
BWA	20	2	20	2	20	2
BOWTIE2	20	2	20	2	20	2

Therefore, to evaluate how trimming and masking affect on the false-negative rate that has enough cases of false-negatives, we sequenced a Hawaiian strain of *C. elegans* and compared our SNPs to Hawaiian SNPs from Wormbase (WS220). This Hawaiian strain has over 112 k SNPs compared to commonly used a Bristol wild-type strain (N2). We could identify 104,297 to 105,355 Hawaiian SNPs from the total of 112,061 Hawaiian SNPs, which were considered as true-positives (Table 4). By setting the difference between the total number of Hawaiian SNPs and the true-positives as false-negatives, we determined the false-negative rates, which were from 6.0% to 6.9%. To determine the statistical significance with respect to preprocessing methods, we performed χ^2^ tests for both BWA and BOWTIE2. The p-values were 0.39 and 0.15, for BWA and BOWTIE2 respectively, which suggests that the differences between preprocessing methods were not statistically significant for these two methods in the false-negative rates.

**Table 4:** **The number of Hawaiian SNPs and the false-negative rate**

**Aligners**	***No quality control***	***Trimming***	***Masking***
BWA	104,858 (6.4%)	105,355 (6.0%)	104,775 (6.5%)
BOWTIE2	104,422 (6.8%)	104,470 (6.8%)	104,297 (6.9%)

### Indels and other considerations

**Figure 6:**
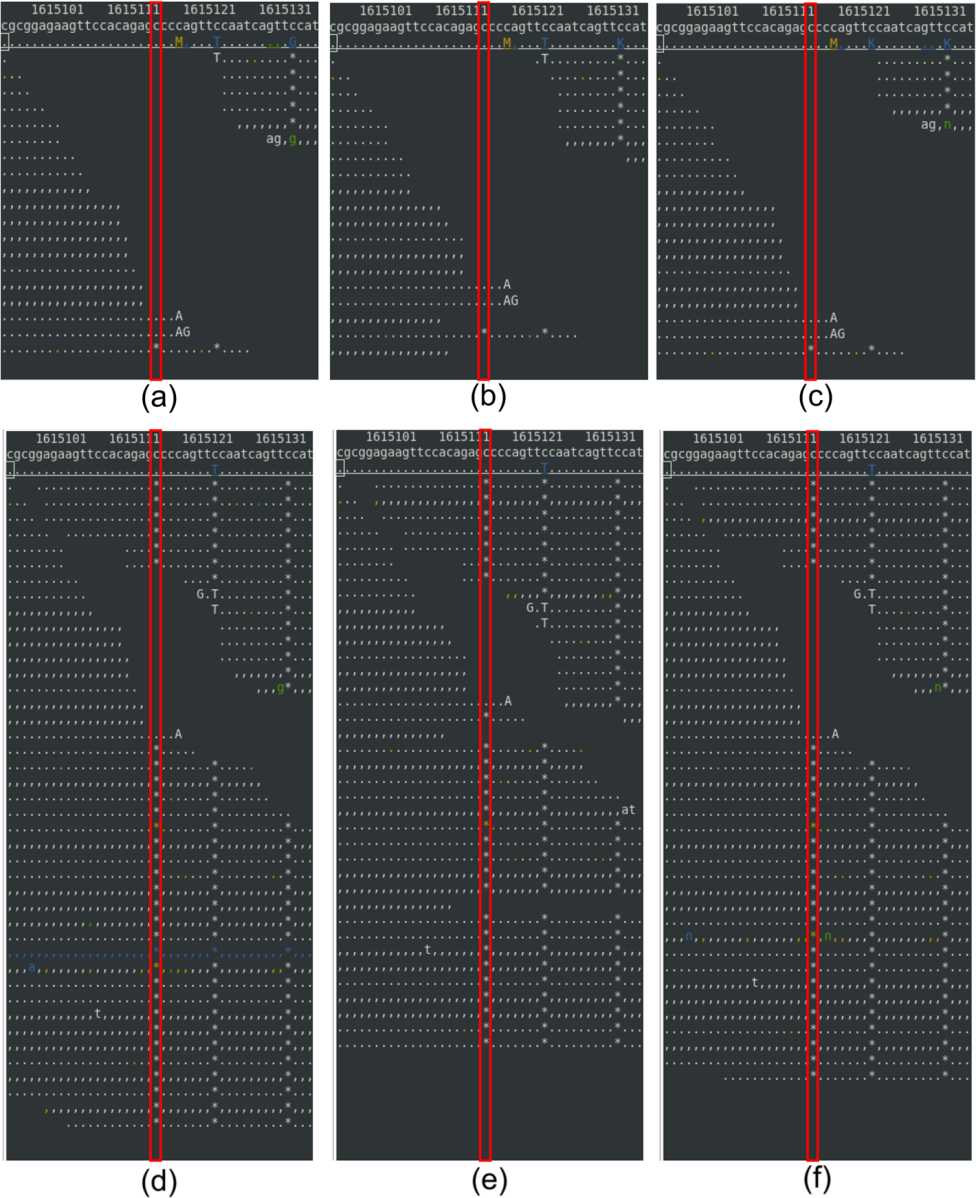
**Alignments for the first false-negative Indel at chromosome X:1,615,135. (a)** no quality control – BWA, **(b)** trimming – BWA, **(c)** masking – BWA, **(d)** no quality control – BOWTIE2, **(e)** trimming – BOWTIE2, and **(f)** masking – BOWTIE2. See the Figure 3 legend for details on coloring. Sanger sequencing identified the deletion of ‘C’.

**Figure 7:**
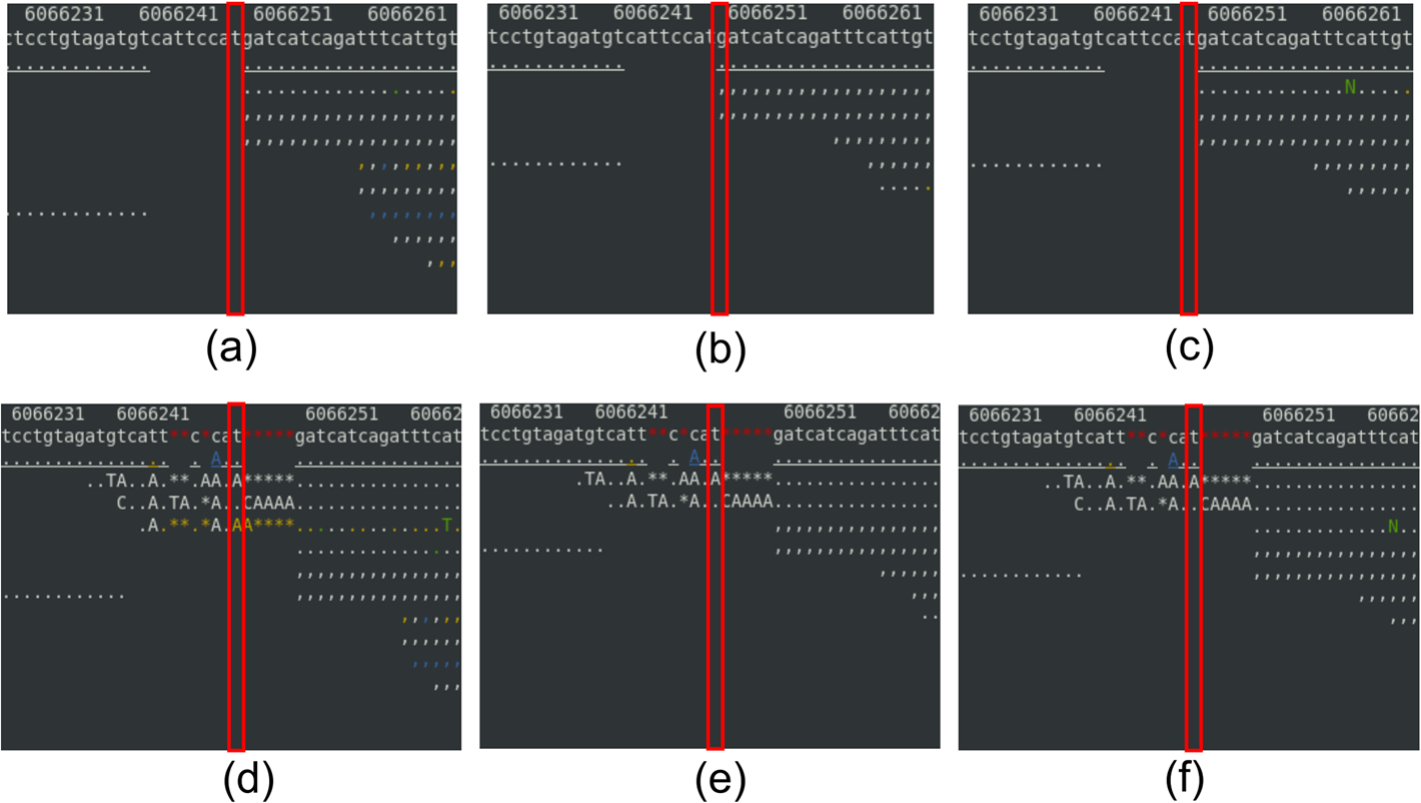
**Alignments for the second false-negative Indel at chromosome I:6,066,249. (a)** no quality control – BWA, **(b)** trimming – BWA, **(c)** masking – BWA, **(d)** no quality control – BOWTIE2, **(e)** trimming – BOWTIE2, and **(f)** masking – BOWTIE2. See the Figure 3 legend for details on coloring.

For a Sanger verified deletion of ‘C’ at chromosome X:1,615,135, BWA and BOWTIE2 aligned the reads, but GATK haplotype caller did not call it as a deletion (Figure 6). For the other Sanger verified insertion, a 21 base insertion of “GATCTCCAATTACAATCAAAA” at chromosome I:6,066,249, it was not called because BWA and BOWTIE2 could not align the reads here (Figure 7). Removal of duplicates did not affect the results for trimming and masking for our Sanger verified SNPs. However, the number of false-positives decreased from 2 to 1 for BWA and from 6 to 3 for BOWTIE2 with no quality control.

To determine if the reduced false positive rate observed with preprocessing using masking or trimming was dependent on the alignment tool, we repeated the analysis using the local alignment mode within BOWTIE2 (option “--local”). The false positive rates did not change for either trimming or masking using the local compared to global alignment modes (Table 5). We did note that in the absence of preprocessing (no quality control), the false positive rate with the local alignment decreased slightly compared to the global alignment, from 7.0% to 5.8%, consistent with more accurate alignment. The reason for only a slight decrease in the false positive rate is because base call errors due to low quality scores prevent alignment even when using the more accurate local alignment mode. Preprocessing steps that remove these errant, low quality base calls improve the false positive rates. Trimming may eliminate one or more high quality base calls in the process and the reduced size of the total read length may increase the chance of misalignment. In contrast, masking saves all the high quality base calls in a read, making it more effective than trimming in reducing the false positive rate.

**Table 5:** **The false positive rates with global alignment vs. local alignment**

	***No quality control***	***Trimming***	***Masking***
**Global alignment**	7.0%	2.3%	0%
**Local alignment**	5.8%	2.3%	0%

## Conclusions

The reason why masking performs better than trimming and no quality control in reducing the false-positive rate is apparent because masking maximizes the information content of a raw read while removing the base calls with low qualities. However, masking did not improve for the false-negative calls due to a low coverage. Therefore, we recommend masking as a preprocessing method to remove low quality base calls in NGS since it reduces the false-positive rate without sacrificing the false-negative rate. Masking could be used effectively in reducing the false-positive rate also for the identification of somatic mutations in cancer screening by RNA-seq. The perl script for masking is available at http://code.google.com/p/subn/.
